# Cebranopadol, a Mixed Opioid Agonist, Reduces Cocaine Self-administration through Nociceptin Opioid and Mu Opioid Receptors

**DOI:** 10.3389/fpsyt.2017.00234

**Published:** 2017-11-13

**Authors:** Qianwei Shen, Yulin Deng, Roberto Ciccocioppo, Nazzareno Cannella

**Affiliations:** ^1^School of Pharmacy, Pharmacology Unit, University of Camerino, Camerino, Italy; ^2^School of Life Sciences, Beijing Institute of Technology, Beijing, China

**Keywords:** cocaine, nociceptin opioid, mu opioid, opioid system, buprenorphine, addiction

## Abstract

Cocaine addiction is a widespread psychiatric condition still waiting for approved efficacious medications. Previous studies suggested that simultaneous activation of nociceptin opioid (NOP) and mu opioid (MOP) receptors could be a successful strategy to treat cocaine addiction, but the paucity of molecules co-activating both receptors with comparable potency has hampered this line of research. Cebranopadol is a non-selective opioid agonist that at nanomolar concentration activates both NOP and MOP receptors and that recently reached phase-III clinical trials for cancer pain treatment. Here, we tested the effect of cebranopadol on cocaine self-administration (SA) in the rat. We found that under a fixed-ratio-5 schedule of reinforcement, cebranopadol (25 and 50 µg/kg) decreased cocaine but not saccharin SA, indicating a specific inhibition of psychostimulant consumption. In addition, cebranopadol (50 µg/kg) decreased the motivation for cocaine as detected by reduction of the break point measured in a progressive-ratio paradigm. Next, we found that cebranopadol retains its effect on cocaine consumption throughout a 7-day chronic treatment, suggesting a lack of tolerance development toward its effect. Finally, we found that only simultaneous blockade of NOP and MOP receptors by concomitant administration of the NOP antagonist SB-612111 (30 mg/kg) and naltrexone (2.5 mg/kg) reversed cebranopadol-induced decrease of cocaine SA, demonstrating that cebranopadol activates both NOP and classical opioid receptors to exert its effect. Our data, together with the fairly advanced clinical development of cebranopadol and its good tolerability profile in humans, indicate that cebranopadol is an appealing candidate for cocaine addiction treatment.

## Introduction

Cocaine is a widely abused illicit drug with a worldwide prevalence of 0.4%, accounting for 14–20 million individuals who have used cocaine in their lifetime (1). Cocaine addiction, for which approved medications are still needed, causes physical, psychiatric, socioeconomic, and judicial problems (2), representing therefore a major social burden. The fourth member of the opioid receptor superfamily, the nociceptin opioid (NOP) receptor, is considered a novel potential target for medication of cocaine addiction due to its anti-rewarding properties and limited side effects of NOP agonists (3–7).

Nociceptin is the endogenous ligand of the NOP that has been proven to inhibit cocaine-induced conditioned place preference (CPP) in the rat (8) and NOP knockout (KO) mice showed higher cocaine-induced CPP than their wild-type counterparts (9). In addition, nociceptin abolished cocaine-elicited psychomotor sensitization in wild type but not NOP KO mice (10). Noteworthy, intracerebroventricular injection of nociceptin failed to induce either place preference or aversion even at a dose as high as 1,000 ng/rat (11). Consistently, NOP agonists have limited abuse liability (12), thus representing promising tools for the treatment of cocaine addiction. Yet, direct evidence of the effect of NOP agonists on cocaine self-administration (SA) is still lacking.

A growing body of evidence indicates that buprenorphine—an NOP receptor and mu opioid (MOP) receptor agonist and kappa (KOP) and delta (DOP) opioid receptors antagonist—reduces cocaine intake partially *via* NOP agonism (13). Buprenorphine combined with low, but not high, doses of naltrexone, maintained its potential to prevent cocaine SA in non-dependent rats (14), which could be explained by residual activation of MOP and NOP (13). Clinical trials demonstrated that only a high dose of buprenorphine (≥16 mg/day) was effective in reducing cocaine use, possibly due to simultaneous activation of NOP as MOP was fully occupied at this dose (3, 6). Similar results were obtained by buprenorphine treatment in alcohol-preferring rats in which high doses decreased alcohol intake through NOP activation, but low doses increased intake by activating MOP (15) However, the limitation of buprenorphine as potential cocaine addiction treatment is owed to its too low affinity to NOP compared with MOP (about 50 times lower) (16), and its related abuse liability (17). Therefore, we hypothesize that compounds with high affinities and strong potency to both NOP and classical opioid receptors may have promising potential for treatment of cocaine addiction with reduced side effects.

Cebranopadol is a compound already tested in both completed and ongoing phase-II and phase-III clinical trials for pain treatment (11, 18). Radioligand binding data revealed sub-nanomolar affinity to both rat and human NOP and MOP 20 times higher than human DOP and 3–4 times higher than human KOP receptors. Cebranopadol is a full agonist for human NOP, MOP, and DOP and partial agonist for KOP receptors (19). Here, we employed cebranopadol to test our hypothesis. In a work concomitantly conducted by others, cebranopadol decreased cocaine fixed ratio 1 (FR1) SA in escalated rats and reduced cue-induced reinstatement of cocaine seeking (20). Here we expanded this investigation by looking at the effect of the drug on cocaine reinforcement and motivation by FR5 and progressive-ratio (PR) SA. Moreover, to validate cebranopadol selectivity for cocaine reinforcement, we tested this compound on saccharin SA. Lastly, the NOP antagonist SB-612111 and the non-selective opioid antagonist naltrexone were employed to determine the pharmacological mechanism by which cebranopadol abolishes cocaine consumption. Rats with stable cocaine SA baseline were also treated chronically with cebranopadol or its vehicle for seven consecutive days to evaluate if the effect of cebranopadol is maintained after repeated treatments.

## Methods and Materials

### Animals

Male Wistar rats (Charler River, Italy) weighing 270–320 g at the beginning of the experiments were used. Animals’ body weight at the time of tests was very similar with groups with an individual difference not larger than 462.4 ± 8.4. Pairs of rats were housed in a room with artificial 12/12 h light/dark cycle (lights off at 8.00 a.m.), at constant temperature (20–22°C) and humidity (45–55%). Food (4RF18, Mucedola, Settimo Milanese, Italy) and water were provided *ad libitum* except during session time. All experiments were conducted during the dark phase of the light/dark cycle. Rats were allowed to acclimate to the housing room for 1 week and were handled three times before any experimental manipulation. All procedures were carried out in accordance with the recommendations of the European Community Council Directive and the National Institutes of Health for the Care and Use of Laboratory Animals and were approved by the University of Camerino Internal Ethical Committee for the Laboratory Animal Protection and Use [(CEAPA) (Protocol no. 3796/12)].

### Drugs

Cocaine hydrochloride and morphine hydrochloride (Sigma, USA) were dissolved in sterile saline. Saccharin (Sigma, Italy) was dissolved in tap water. Cebranopadol (Biochempartner Co., Ltd., China) for operant tests was diluted to a fine suspension with 5% DMSO and 95% glucose (5%) and administered *per os* (p.o.) by gavage. We chose p.o. administration to mimic the most common administration route in human. Cebranopadol for place conditioning was dissolved in 10% DMSO + 5% Cremophor EL + 85% saline and administered intraperitoneally (i.p.). For CPP, we used the i.p. route to facilitate the rapid absorption of the drug that is important to achieve the expression of place conditioning. Cebranopadol doses were selected based on previously reported p.o. ED_50_ value. Previous work demonstrated that pharmacological efficacy of cebranopadol is maintained for least 9 h (19). The selective NOP antagonist SB-612111 was obtained from Tocris (USA) and was dissolved in 1-M H_3_PO_4_ in distilled water (1:1), the opioid receptors antagonist naltrexone (Sigma, USA) was dissolved in distilled water. Evidence demonstrates that the doses chosen for the two antagonists result in an appropriate brain bioavailability and significant central nervous system effects (21, 22). Pharmacokinetic data on naltrexone and SB-612111 interactions are not available.

### Catheter Implantation

Animals were anesthetized by intramuscular injection of 100–150 µL of a solution containing tiletamine cloridrate (58.17 mg/mL) and zolazepam chlorohydrate (57.5 mg/mL). For IV surgery, incisions were made to expose the right jugular vein. A catheter made from micro-renathane tubing (ID = 0.020′′, OD = 0.037′′) was subcutaneously positioned between the vein and the back. After insertion into the vein, the proximal end of the catheter was anchored to the muscles underlying the vein with surgical silk. The distal end of the catheter was attached to a stainless steel cannula bent at an angle of 90°. The cannula was inserted into a support made by dental cement on the back of the animals and was covered with a plastic cap. Immediately after surgery, rats were treated intramuscularly with 200 µL of enrofloxacin (50 mg/mL, Baytril, Germany).

Rats were allowed to recover 1 week before SA training. Catheters were flushed with 100 μL/rat of heparinized saline (20 UI/mL) prior to each SA session, when appropriate 0.5 mg/mL of enrofloxacin was added to the flushing solution. Catheter patency was confirmed by intravenous injection of 150 μL/rat of pentothal sodium (25 mg/mL, Intervet, Italy) at the end of experimental procedures.

### SA Apparatus

The SA stations consisted of operant conditioning chambers (Med Associate Inc.) enclosed in sound attenuating and ventilated environmental cubicles. Each chamber was equipped with two retractable levers located in the front panel of the chamber. Before the beginning of the session, an infusion pump delivering cocaine was connected by a polyethylene tube to the catheter. The infusion pump was activated by responses on the right (active) lever, while responses on the left (inactive) lever were recorded but did not result in any programmed consequences. Activation of the pump resulted in delivery of 0.1 mL of fluid. An IBM compatible computer controlled the delivery of cocaine solution and recording of the behavioral data.

### Effect of Acute Cebranopadol on Cocaine SA

#### Fixed Ratio

Rats (*n* = 8) were initially trained for 2-h daily cocaine SA sessions under FR1 schedule of reinforcement for 10 days, then reinforcement schedule was increased to FR5 until stable baseline of responding (<10% variation for three consecutive days) was reached. Following each cocaine infusion (0.25 mg/0.1-mL intravenous), a 20-s time out (TO) period was presented during which responses at the active lever had no programmed consequences. Cebranopadol (0, 25, 50 µg/kg p.o. 1 h before test) was tested in a Latin-square counterbalanced design. At least, a 3-day interval during which cocaine SA baseline was reestablished was allowed between drug tests.

#### Progressive Ratio

For PR experiments, a new cohort of rats (*n* = 8) was trained to cocaine SA as described above. After training, the effect of cebranopadol on cocaine SA under PR schedule of reinforcement was tested. During PR sessions, the response requirements necessary to receive a single cocaine dose increased according to the following scale: 5, 11, 18, 26, 35, 45, 56, 68, 82, 98, 116, 136, 158, 182, 208, 236, 268, and 304. PR session stopped after 6 h or if the required ratio was not achieved within 1 h, whichever came first. The breakpoint (BP) corresponding to the last ratio completed was used as a measure of motivation. One hour before test, rats (*n* = 8) were treated with cebranopadol (0, 25, 50 µg/kg p.o.) in a Latin-square counterbalanced design. Drug treatment was performed every fourth day. Baseline FR5 cocaine SA was reestablished between PR tests.

### Effect of Acute Cebranopadol on Saccharin SA

Another group of rats (*n* = 8) was used to test the effect of cebranopadol on oral saccharin (0.2% w/v) SA. Rats were trained to 30-min daily sessions under an FR1 schedule of reinforcement until stable lever pressing baseline was reached. At this point, the effect of cebranopadol (0, 25, 50 µg/kg p.o.) was tested in a Latin-square counterbalanced design. Drug treatment was performed every fourth day, 1 h before test. Baseline FR1 saccharin SA was reestablished between tests. We used FR1 because we expected, based on our previous experience, that this schedule of reinforcement would produce in 30 min of saccharin SA—a range of lever responses comparable to the initial phase (30–60 min) of cocaine SA in FR5.

### Effect of Chronic Treatment with Cebranopadol on Cocaine SA

The effect of repeated cebranopadol administrations was tested to evaluate if the effect of the drug on cocaine intake was maintained following chronic treatment. For seven consecutive days, two groups of rats (*n* = 7–8/group) with similar cocaine SA baseline were treated with cebranopadol (25 µg/kg p.o.) or its vehicle given 1 h before initiating the 2-h SA sessions under FR5.

### Effect of SB-612111 and Naltrexone on Cebranopadol-Induced Inhibition of Cocaine SA

To explore the mechanisms of action of cebranopadol, we tested the effect of the selective NOP antagonist SB-612111 (30 mg/kg p.o.), the MOP/KOP/DOP antagonist naltrexone (Nal, 2.5 mg/kg i.p.) or their combination on cebranopadol-induced inhibition of cocaine SA (23, 24). One hour before session, rats (*n* = 10) received cebranopadol (50 µg/kg p.o.) or its vehicle alone or in combination with SB-612111, naltrexone or both antagonists together. Rats were subjected to all treatment conditions in a Latin-square design. An interval of at least 3 days during which cocaine SA baseline was reestablished was allowed between drug tests.

### Cebranopadol-Induced Place Conditioning

To test whether cebranopadol has rewarding effects *per se*, we measured the place conditioning induced by the drug in a two compartment apparatus using an unbiased schedule of conditioning. The first day rats (*n* = 8/group) were allowed to explore the entire apparatus for 15 min (preconditioning) to confirm lack of innate preference for one of the two compartments. From the second day, animals underwent one 25-min conditioning session per day for 6 days. Cebranopadol groups (10 and 50 µg/kg i.p.) received three cebranopadol treatments in one compartment or three vehicle treatments in the opposite compartment every other day. The control group received vehicle injections in both compartments. Treatments were balanced across compartments and days. On the seventh day, drug free animals were allowed to explore the entire apparatus for 15 min. An operator unaware of treatment conditions recorded time spent in each compartment scoring test video clips. A rat was considered to have entered a compartment when all its four paws stepped inside. Rats received treatments 15 min before session.

We treated a group of rats (*n* = 7) with morphine (5 mg/kg i.p.) with the same conditioning schedule as a positive control to check whether our protocol is suitable to assess opioid-induced place conditioning. Place-conditioning protocol was designed to maximize drug effect based on meta-analysis data (25).

### Statistical Analysis

The effect of acute cebranopadol on cocaine and saccharin SA, as well as the effect of SB-612111 and naltrexone on cebranopadol-induced reduction of cocaine infusions, was analyzed by one-way analysis of variance (ANOVA) with treatment as a repeated measure. The effect of chronic cebranopadol on cocaine SA was analyzed by two-way ANOVA with one factor between (treatment) and one factor within (time). Inactive-level responses were separately analyzed and used as an additional measure to monitor the specificity of drug effects. The Newman–Keuls test was used for *post hoc* analysis when appropriate.

In the CPP experiment, to verify that our protocol was unbiased, we used the *t*-test for dependent samples to compare the time rats spent in each compartment during the preconditioning test. For the control group, absence of side preference was tested also on the test day.

Preference score was defined as the time spent in the drug-paired compartment minus the time spent in the vehicle-paired compartment. For the control group, which received vehicle in both compartments, the “drug-paired compartment” was randomly assigned before test. We analyzed morphine place conditioning by *t*-test for independent groups (vehicle vs. morphine). We analyzed cebranopadol place conditioning by one-way ANOVA with groups as independent factors (cebranopadol: 0, 10, and 50 µg/kg).

Results were expressed as mean ± SEM and statistical significance was set at *p* < 0.05.

## Results

### Acute Cebranopadol Selectively Reduced Cocaine SA

**Figure 1:**
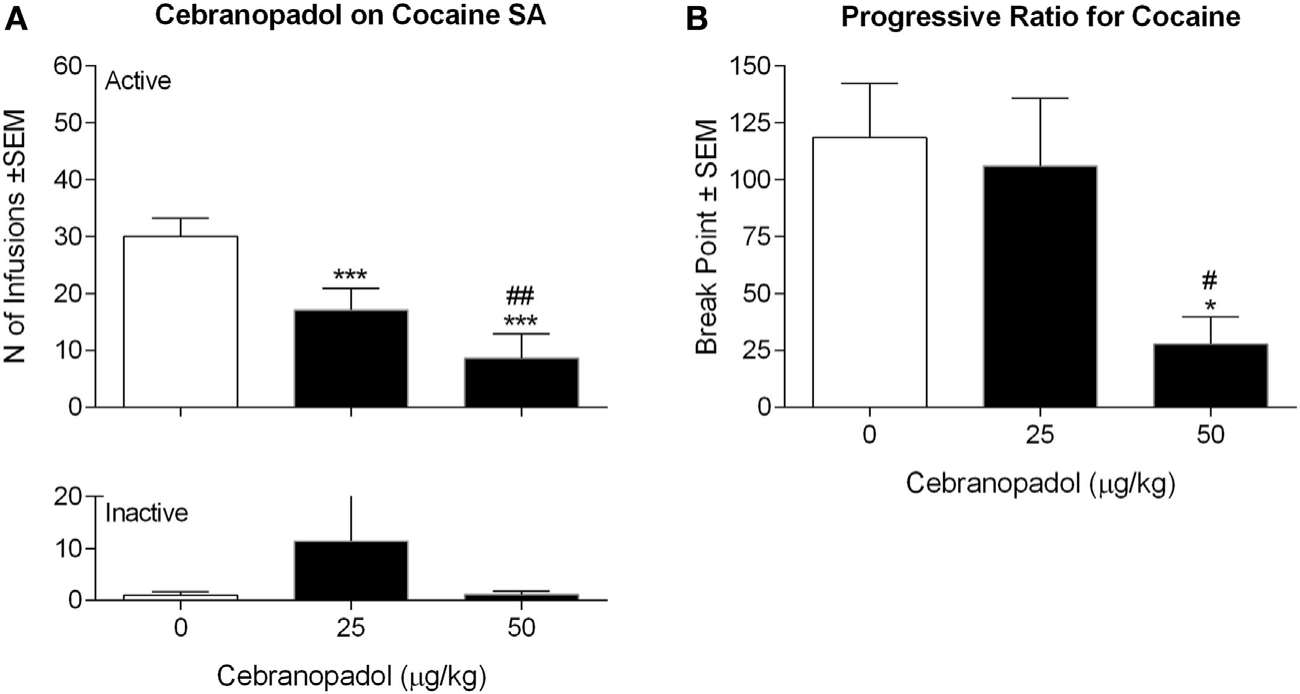
Effect of cebranopadol on cocaine SA under fixed **(A)** and progressive **(B)** ratio of responses. **(A)** Both doses of cebranopadol decreased the number of cocaine infusions earned (top panel); the effect of cebranopadol was specific, as inactive lever responses were not affected by treatments (bottom panel). **(B)** When cebranopadol was tested on motivation for cocaine expressed as breakpoint reached in SA session under a progressive-ratio schedule of reinforcement, the dose of 50 µg/kg, but not 25 µg/kg, decreased the break point for cocaine. Values are presented as mean ± SEM. Statistical differences: *p < 0.05 and ***p < 0.001 vs. vehicle; ^#^*p* < 0.05 and ^##^*p* < 0.01 vs. 25 µg/kg. SA, self-administration.

Rats rapidly acquired a stable baseline of cocaine responding under FR5 contingency. ANOVA on the effect of cebranopadol on cocaine SA revealed an overall effect of doses [*F*(2, 7) = 43.1069, *p* < 0.00001] (Figure 1A). *Post hoc* analysis revealed a significant decrease in the number of cocaine reinforced responding at both doses of cebranopadol tested (*p* < 0.001). Inactive lever pressing was very low and was not affected by drug treatment [*F*(2, 7) = 0.9143, *p* > 0.05].

When the effect of cebranopadol on motivation for cocaine was evaluated under PR contingency, overall ANOVA of the break points found an overall effect of doses [*F*(2, 7) = 6.915, *p* < 0.01]. As shown in Figure 1B, *post hoc* tests indicated a significant reduction (*p* < 0.05) of break point following administration of the highest dose (50 µg/kg) of the drug.

### Acute Cebranopadol Selectively Increased Saccharin SA

**Figure 2:**
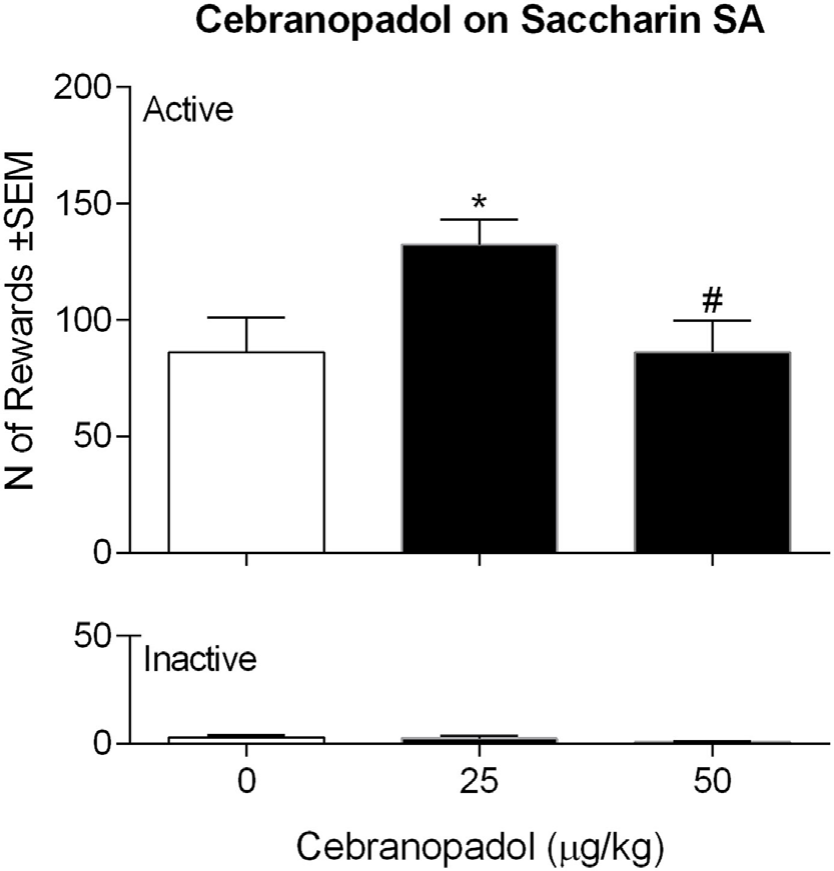
Effect of cebranopadol on saccharin SA. The dose of 25 µg/kg, but not 50 µg/kg, increased the number of saccharin rewards self-administered (top panel). Inactive lever response was not affected by cebranopadol treatments (bottom panel). Data are presented as mean ± SEM. Statistical differences: *p < 0.05 vs. vehicle; ^#^*p* < 0.05 vs. 25 µg/kg. SA, self-administration.

Rats rapidly acquired saccharin SA under FR1 contingency. ANOVA on the effect of cebranopadol on saccharin SA revealed an overall effect of doses [*F*(2, 7) = 4.859, *p* < 0.05]. As shown in Figure 2, *post hoc* analysis showed a significant increase (*p* < 0.05) in saccharin reinforcement earned following administration of 25 µg/kg of cebranopadol. At a higher dose (50 µg/kg), the drug did not modify saccharin SA. Inactive lever pressing was very low and was not affected by drug treatment [*F*(2, 7) = 2.223, *p* = 0.1451].

### Reduction of Cocaine SA Maintained Following Chronic Administration of Cebranopadol

**Figure 3:**
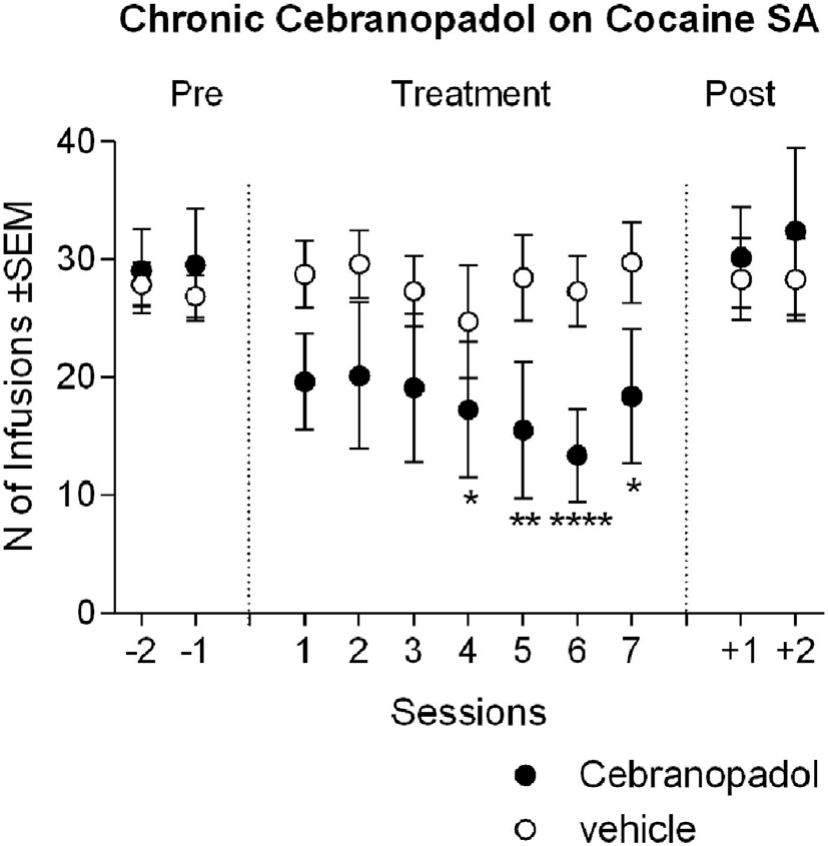
Chronic cebranopadol treatment on cocaine self-administration. Two groups of rats with similar baseline of cocaine intake received cebranopadol (25 µg/kg) or its vehicle for seven consecutive sessions. The number of cocaine infusions earned by the cebranopadol-treated group decreased during treatment in respect to baseline, and returned to pretreatment level from the first day posttreatment. In the vehicle-treated group, cocaine self-administration remained stable throughout the experiment. Data are presented as mean ± SEM and show the last 2 days of pretreatment, 7 days of treatment, and 2 days of posttreatment. Statistical differences: *p < 0.05, **p < 0.01, and ****p < 0.0001 vs. the last day pretreatment.

Analysis of variance of cocaine infusion earned during chronic cebranopadol treatment revealed an overall effect of days [*F*(10, 13) = 4.74, *p* < 0.001], while overall effect of groups was not significant [*F*(1, 13) = 1.00, *p* = 0.334]. However, there was a significant treatment-by-group interaction [*F*(10, 130) = 4.51, *p* < 0.001]. *Post hoc* tests indicated that there were no significant differences between the first three treatment days and pretreatment SA level, whereas a significant inhibition in the number of cocaine reinforced responding was detected from days 4 to 7 (Figure 3). When treatment was discontinued, cocaine SA returned to pretreatment levels. Analysis of the inactive lever responding found no effect of group [*F*(1, 13) = 0.48, *p* > 0.05], days [*F*(10, 13) = 0.96, *p* > 0.05], or group-by-day interaction [*F*(10, 130) = 1.42, *p* > 0.05]. This result seems in part to contrast with the effect of acute administration of cebranopadol, where the dose of 25 µg/kg reduced cocaine SA. This discrepancy is probably due to the different treatment design, Latin-square rotation of each dose in the acute experiment and between subjects in the chronic one. To confirm that this apparent discrepancy was due to the experimental design, using the *t*-test analysis for dependent samples, we compared the first drug test session with the last cocaine-training day. As expected, results showed a significant [*t*(7) = 4.05; *p* < 0.01] reduction of cocaine SA following cebranopadol.

### Effect of SB-612111 and Naltrexone on Cebranopadol-Induced Inhibition of Cocaine SA

**Figure 4:**
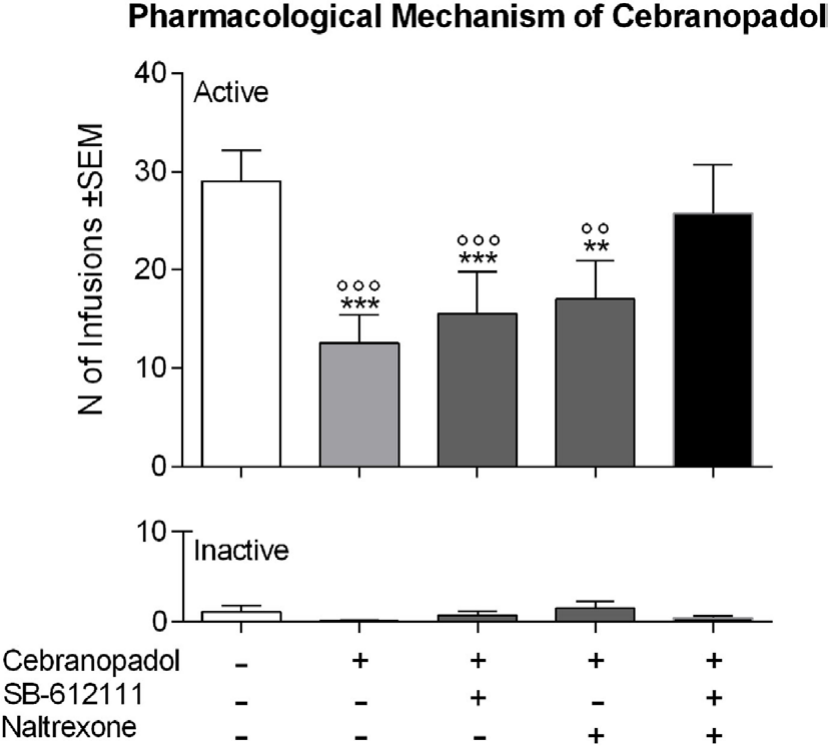
Pharmacological mechanism by which cebranopadol decreases cocaine self-administration. 50 µg/kg of cebranopadol reduced the number of cocaine infusions self-administered. The effect of cebranopadol was counteracted by coadministration of the NOP antagonist SB-612111 (30 mg/kg) and the MOP antagonist naltrexone (2.5 mg/kg) or their vehicles. Only simultaneous administration of SB-612111 and naltrexone could fully counteract cebranopadol effect and reestablished cocaine intake to control level. Neither SB-612111 nor naltrexone given alone altered cebranopadol effect (top panel). The effects of treatments were specific for cocaine seeking as inactive lever responses were not affected by treatments (bottom panel). Administration of vehicle for each compound is indicated by the “–” mark, while administration of the active agent is indicated by the “+” mark. Data are presented as mean ± SEM. Statistical differences: **p < 0.01 and ***p < 0.001 vs. control treatment; ^°°^*p* < 0.01 and ^°°°^*p* < 0.01 vs. cebranopadol + SB-612111 + naltrexone treatment. MOP, mu opioid; NOP, nociceptin opioid.

To investigate the pharmacological mechanism by which cebranopadol inhibits cocaine intake, we tested the effect of SB-612111, a selective NOP antagonist, and of the MOP/KOP/DOP antagonist naltrexone, on cebranopadol-induced inhibition of cocaine SA. When number of infusions was analyzed, ANOVA revealed an overall effect of treatment [*F*(4, 8) = 8.147, *p* < 0.001]. As shown in Figure 4, Newman–Keuls *post hoc* test indicated, as expected, that 50 µg/kg cebranopadol significantly reduced the number of cocaine reinforced responding (*p* < 0.001). Neither SB-612111 nor naltrexone alone were able to antagonize cebranopadol effect. In contrast, when SB-612111 and naltrexone were coadministered they completely reversed cebranopadol-induced reduction of cocaine SA (*p* < 0.01). Inactive lever responding was very low, and as shown by overall ANOVA, was not affected by treatments [*F*(4, 8) = 1.152, *p* > 0.05].

### Cebranopadol Not Inducing Place Conditioning

**Figure 5:**
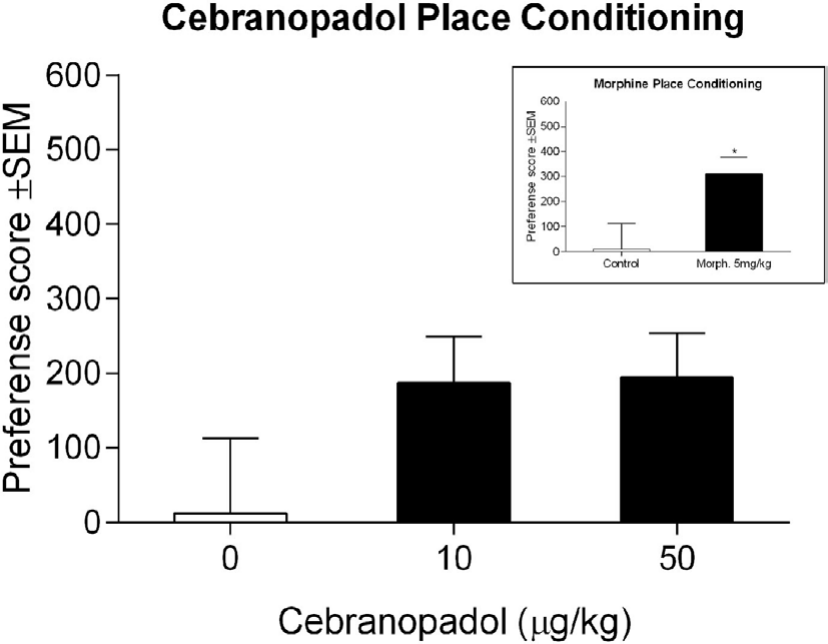
Effect of cebranopadol on place-conditioning test. Neither of the two doses of cebranopadol induced place conditioning, although a trend to spend more time in the drug-paired compartment could be observed. *Inset*: morphine induced a clear place preference. Morphine’s preference score was 1.7-fold higher than cebranopadol’s. Preference score is reported in seconds. Data are presented as mean ± SEM. *p < 0.05 vs. control group.

Control rats did not show preference for either of the compartments, demonstrating that place conditioning was performed in unbiased conditions [*t*(8) = 0.44; *p* = 0.67]. Morphine-treated rats showed marked development of CPP with a significant preference score [*t*(13) = −2.33; *p* < 0.05], demonstrating that our protocol was suitable to test place preference induced by drugs targeting the opioid system (Figure 5 inset). For cebranopadol, ANOVA revealed no overall effect of groups [*F*(2.21) = 1.83; *p* > 0.05], indicating that cebranopadol did not induce significant place-conditioning effects (Figure 5).

## Discussion

Results showed that cebranopadol significantly reduced cocaine SA under fixed and progressive ratio schedule of reinforcements, indicating reduced motivation for cocaine following drug treatment. Our data are in good agreement with work concomitantly conducted by de Guglielmo et al. (20), showing that cebranopadol reduces cocaine SA in rats after escalation of cocaine intake in 6-h sessions. The effect of cebranopadol was substance-specific because when we tested cebranopadol on saccharin SA, we found a slight increase in lever pressing at the lowest dose and no changes at the highest dose. A similar finding was described by de Guglielmo et al. (20) who attributed that using sweet condensed milk as reinforcer demonstrated the same behavioral pattern. The tendency of cebranopadol to increase the consumption of sweet solutions is consistent with its ability to activate MOP receptors. In fact, sweet intake is markedly enhanced following activation of this opioid receptor by morphine, DAMGO or other selective agonists (26–29). In addition, the fact that cebranopadol does not decrease, or even increases, responses for natural reinforcers demonstrates that the effect on cocaine was not secondary to sedation or to general disruption of motor activity. In the chronic study, cebranopadol slightly decreased cocaine SA at the beginning of the treatment; the effect progressively increased following repeated administrations and became significant after 4 days. This finding supports the feasibility of chronic drug administration as a therapeutic strategy for cocaine addiction. We also demonstrated that the inhibitory effect of cebranopadol on cocaine SA is blocked by coadministration of SB-612111 and naltrexone, while compounds alone did not affect the efficacy of cebranopadol. This indicates that the effect of cebranopadol on cocaine SA is mediated by two possibly independent mechanisms involving NOP and classical opioid receptors. Only simultaneous blockade of these two pathways warrants full inhibition of cebranopadol effects on cocaine. Finally, in a place-conditioning test, we found that in contrast with morphine, cebranopadol did not elicit significant expression of place preference, although a trend to an effect was detected (30, 31). Additional studies (i.e., drug discrimination, operant intravenous SA) will have to be carried out to thoroughly evaluate the abuse potential of this compound.

Cebranopadol is a high-affinity agonist at both MOP and NOP receptors and is almost equipotent in activating both receptors. At higher concentrations, it also activates KOP and DOP receptors (19). The pharmacological profile of cebranopadol is partially mimicked by buprenorphine, a well-characterized compound that acts as a high-affinity partial agonist at MOP and low-affinity NOP agonist, but is an antagonist at KOP and DOP receptors (32). Like cebranopadol, buprenorphine reduced cocaine intake in the rat (13, 33, 34). This effect was confirmed also in rhesus monkeys (35) and in heroin-dependent patients co-abusing cocaine (36). Importantly, clinical data demonstrated that reduction of cocaine consumption by buprenorphine is independent of the effect on heroin intake and occurs only at doses higher than 24 mg/kg (36). This dose is substantially higher (>16 mg/day) compared with those needed to fully occupy MOP, and most likely KOP receptors, as the drug shows equivalent affinity for both receptors (32, 36–39). Based on these receptor affinity profiles, we argued that the inhibitory effect of buprenorphine on cocaine consumption was mediated by activation of NOP, which occurs at substantially higher drug doses (15, 32, 40, 41). Contrary to our expectation, we found that selective blockade of NOP did not prevent the effect of buprenorphine. However, the effect was fully prevented if NOP and MOP receptors were simultaneously blocked (13). Based on this observation, we concluded that buprenorphine reduces cocaine consumption with mechanisms involving both NOP and MOP receptors. Here we demonstrated that, cebranopadol despite acting as a KOP and DOP agonist, whereas buprenorphine is an antagonist, also attenuated cocaine intake. Based on these data, it is tempting to speculate that KOP-dependent mechanisms might not be relevant in mediating the effects of these drugs on cocaine consumption.

In addition to NOP, MOP, and KOP receptors, although at higher concentrations, cebranopadol binds also to DOP (19). Based on present results, we cannot fully exclude the possible involvement of this opioid receptor subtype in mediating the effect of cebranopadol on cocaine. However, this hypothesis is discouraged by the lower affinity of this compound for DOP and by the fact that psychostimulant consumption is blocked by DOP antagonism (42). Cebranopadol is at phase-III stage of development for the treatment of pain, and toxicological data demonstrated that it is well tolerated in humans (11). Its safety profile was confirmed by studies in rodents showing that, contrary to classic opioid analgesics, cebranopadol did not produce respiratory depression and impairment of motor coordination at pharmacological effective doses (19). The opportunity to test this compound in cocaine addicts is therefore relatively close.

Theoretically, the ability of cebranopadol to act as KOP agonist could rise concerns on the use of this compound in drug addicts. In fact, previous studies have shown that activation of KOP receptors is associated with dysphoria, exacerbation of anxiety, and enhanced motivation to take drugs of abuse (43–50). For instance, it has been shown that activation of KOP, recruiting stress response mechanisms, is able to reinstate extinguished CPP for cocaine in the rat (51, 52). Moreover, in monkeys trained to self-administer cocaine, the administration of selective KOP agonists facilitates reinstatement of drug seeking and enhances preference for cocaine if animals are trained to a concurrent-choice schedule—choice between food or cocaine (45, 53). Here we showed that in the place-conditioning test cebranopadol did not evoke aversion and produced a trend to a preference probably mediated by its ability to activate MOP receptors. Hence, unlike selective KOP agonist, cebranopadol appeared to be devoid of negative affective properties in the rat (54–58).

Attenuation of drug effect following repeated administration is always a concern when chronic treatment is required in the clinic setting. Interestingly, an earlier study exploring the analgesic effect of cebranopadol revealed that the anti-allodynic effect of the drug is maintained for substantially longer time compared with chronic morphine, which may reflect reduced propensity to produce pharmacological tolerance (19). Here, we further explored this phenomenon by investigating the effect of 1-week treatment with crebranopadol on cocaine SA. Results demonstrated that the effect improved along the 7 days of drug administration, with an important trend toward an increase at the end of treatment. Classically, repeated administrations of opioid agonists lead to rapid development of tolerance; using cebranopadol here this effect did not occur (59, 60). In contrast with other opioidergic agents, buprenorphine shows reduced propensity to evoke tolerance. For instance, reduction of cocaine SA in monkeys was fully retained for a period as long as 120 days (35). Based on these observations, it is tempting to speculate that the pharmacological profile of cebranopadol—mimicking buprenorphine properties, but showing even higher safety and possibly lower abuse liability profile—may be suitable for a chronic therapy in cocaine addicts.

The reasons why molecules with mixed MOP/NOP agonist profile have low tendency to produce tolerance remain unclear at present and, to some extent, contrast with older data, suggesting that activation of central nociceptin system facilitates analgesic tolerance elicited by chronic morphine (61). To explain this phenomenon, further studies should analyze the molecular mechanisms and, possibly, the intracellular signaling pathways that are engaged when MOP and NOP receptors, which are largely co-expressed in the brain, are co-activated (62). Interestingly, cebranopadol differently from other agonists shows low ability to stimulate the opioid receptors-β-arrestin pathway that is typically involved in receptor internalization or desensitization (63, 64).

Altogether these results demonstrate that cebranopadol, in addition to its already known analgesic profile, shows efficacy in attenuating cocaine consumption. This effect is specific and does not generalize toward other ingesta (i.e., saccharin). Moreover, present findings demonstrate that NOP and classical opioid receptor pathways are both involved in mediating these actions of cebranopadol on cocaine. One limitation of the study is that cebranopadol was tested against a fixed dose of cocaine. This does not allow to draw conclusions on whether the effect of this opioid panagonist depends upon its ability to shift the classically U-shaped dose response curve of cocaine toward the right (reflecting attenuation of reward) or toward the left (reflecting potentiation of reward). In recent years, despite significant efforts made to develop new medications for the treatment of cocaine addiction, very little is available for clinical use. The fairly advanced clinical development of cebranopadol makes this compound an ideal candidate for an immediate clinical investigation in cocaine-dependent patients.

## Ethics Statement

All procedures were carried out in accordance with the European Community Council directive and the National Institutes of Health guidelines for the care and use of laboratory animals.

## Author Contributions

QS performed self-administration experiments, analyzed data, and wrote the manuscript. YD contributed to design of the experiment and provided a critical reading. RC wrote the manuscript. NC run place conditioning, analyzed data, and wrote the manuscript.

## Conflict of Interest Statement

The authors declare that they have no competing or conflicting interest and no financial disclosures to include. The reviewers LR-K and MS and handling editor declared their shared affiliation.

## References

[1] UNODC. In: Onu, editor. World Drug Report 2015. (2015).

[2] Karila L Gorelick D Weinstein A Noble F Benyamina A Coscas S New treatments for cocaine dependence: a focused review. Int J Neuropsychopharmacol (2008) 11:425–38. 10.1017/S1461145707008097 17927843

[3] Mannelli P. Agonist-antagonist combinations in opioid dependence: a translational approach. Dipend Patologiche (2010) 5:17. 22448305 PMC3311161

[4] Zaveri NT. The Nociceptin/Orphanin FQ receptor (NOP) as a target for drug abuse medications. Curr Top Med Chem (2011) 11:1151–6. 10.2174/156802611795371341 21050175 PMC3899399

[5] Toll L. The use of bifunctional NOP/Mu and NOP receptor selective compounds for the treatment of pain, drug abuse, and psychiatric disorders. Curr Pharm Des (2013) 19:7451–60. 10.1124/pr.114.009209 23448477

[6] Witkin JM Statnick MA Rorick-Kehn LM Pintar JE Ansonoff M Chen YY The biology of Nociceptin/Orphanin FQ (N/OFQ) related to obesity, stress, anxiety, mood, and drug dependence. Pharmacol Ther (2014) 141:283–99. 10.1016/j.pharmthera.2013.10.011 24189487 PMC5098338

[7] Lutfy K Zaveri NT. The nociceptin receptor as an emerging molecular target for cocaine addiction. Prog Mol Biol Transl Sci (2016) 137:149–81. 10.1016/bs.pmbts.2015.10.003 26810001 PMC5555398

[8] Kotlinska J Wichmann J Legowska A Rolka K Silberring J Orphanin FQ/nociceptin but not Ro 65-6570 inhibits the expression of cocaine-induced conditioned place preference. Behav Pharmacol (2002) 13:229–35. 10.1097/00008877-200205000-00006 12122313

[9] Marquez P Nguyen AT Hamid A Lutfy K. The endogenous OFQ/N/ORL-1 receptor system regulates the rewarding effects of acute cocaine. Neuropharmacology (2008) 54:564–8. 10.1016/j.neuropharm.2007.11.003 18082848 PMC2276976

[10] Bebawy D Marquez P Samboul S Parikh D Hamid A Lutfy K Orphanin FQ/nociceptin not only blocks but also reverses behavioral adaptive changes induced by repeated cocaine in mice. Biol Psychiatry (2010) 68:223–30. 10.1016/j.biopsych.2010.02.010 20359694 PMC2896563

[11] Lambert DG Bird MF Rowbotham DJ Cebranopadol: a first in-class example of a Nociceptin/Orphanin FQ receptor and opioid receptor agonist. Br J Anaesth (2015) 114:364–6. 10.1093/bja/aeu332 25248647

[12] Lin AP Ko M-C The therapeutic potential of Nociceptin/Orphanin FQ receptor agonists as analgesics without abuse liability. ACS Chem Neurosci (2012) 4:214–24. 10.1021/cn300124f 23421672 PMC3582300

[13] Kallupi M Shen Q De Guglielmo G Yasuda D Journigan BV Zaveri JT Reduction of cocaine consumption by buprenorphine requires concomitant activation of NOP and MOP opioid receptor. Addict Biol (2017). 10.1111/adb.12513 PMC5740020 28635181

[14] Wee S Vendruscolo LF Misra KK Schlosburg JE Koob GF A combination of buprenorphine and naltrexone blocks compulsive cocaine intake in rodents without producing dependence. Sci Transl Med (2012) 4(146):146ra110 10.1126/scitranslmed.3003948 PMC3448552 22875830

[15] Ciccocioppo R Economidou D Rimondini R Sommer W Massi M Heilig M. Buprenorphine reduces alcohol drinking through activation of the Nociceptin/Orphanin FQ-NOP receptor system. Biol Psychiatry (2007) 61:4–12. 10.1016/j.biopsych.2006.01.006 16533497 PMC3035814

[16] Khroyan TV Polgar WE Cami-Kobeci G Husbands SM Zaveri NT Toll L The first universal opioid ligand, (2S)-2- (5R,6R,7R,14S)-N-cyclopropylmethyl-4,5-epoxy-6,14-ethano-3-hydroxy-6-methoxymorphinan-7-yl -3,3-dimethylpentan-2-ol (BU08028): characterization of the in vitro profile and in vivo behavioral effects in mouse models of acute pain and cocaine-induced reward. J Pharmacol Exp Ther (2011) 336:952–61. 10.1124/jpet.110.175620 21177476 PMC3061529

[17] Lavonas EJ Severtson SG Martinez EM Bucher-Bartelson B Le Lait M-C Green JL Abuse and diversion of buprenorphine sublingual tablets and film. J Subst Abuse Treat (2014) 47:27–34. 10.1016/j.jsat.2014.02.003 24680219

[18] Schunk S Linz K Hinze C Frormann S Oberboersch S Sundermann B Discovery of a potent analgesic NOP and opioid receptor agonist: cebranopadol. ACS Med Chem Lett (2014) 5:857–62. 10.1021/ml500117c 25147603 PMC4137374

[19] Linz K Christoph T Tzschentke TM Koch T Schiene K Gautrois M Cebranopadol: a novel potent analgesic Nociceptin/Orphanin FQ peptide and opioid receptor agonists. J Pharmacol Exp Ther (2014) 349:535–48. 10.1124/jpet.114.213694 24713140

[20] de Guglielmo G Matzeu A Kononoff J Mattioni J Martin-Fardon R George O. Cebranopadol blocks the escalation of cocaine intake and conditioned reinstatement of cocaine seeking in rats. J Pharmacol Exp Ther (2017) 362:378–84. 10.1124/jpet.117.241042 28645915 PMC5539589

[21] Burattini C Mcgeehan AJ Griffin WC III Gass JT Kinder JR Janak PH A microdialysis study of extracellular levels of acamprosate and naltrexone in the rat brain following acute and repeated administration. Addict Biol (2008) 13:70–9. 10.1111/j.1369-1600.2008.00097.x 18269381

[22] Cippitelli A Schoch J Debevec G Brunori G Zaveri NT Toll L. A key role for the N/OFQ-NOP receptor system in modulating nicotine taking in a model of nicotine and alcohol co-administration. Sci Rep (2016) 6:26594. 10.1038/srep26594 27199205 PMC4873733

[23] Zhang Y Elbegdorj O Yuan YY Beletskaya IO Selley DE. Opioid receptor selectivity profile change *via* isosterism for 14-O-substituted naltrexone derivatives. Bioorg Med Chem Lett (2013) 23:3719–22. 10.1016/j.bmcl.2013.05.027 23721804 PMC3690547

[24] Zaratin PF Petrone G Sbacchi M Garnier M Fossati C Petrillo P Modification of nociception and morphine tolerance by the selective opiate receptor-like orphan receptor antagonist (-)-cis-1-methyl-7- 4-(2,6-dichlorophenyl)piperidin-1-yl methyl -6,7,8, 9-tetrahydro-5H-benzocyclohepten-5-ol (SB-612111). J Pharmacol Exp Ther (2004) 308:454–61. 10.1124/jpet.103.055848 14593080

[25] Bardo MT Rowlett JK Harris MJ. Conditioned place preference using opiate and stimulant drugs: a meta-analysis. Neurosci Biobehav Rev (1995) 19:39–51. 10.1016/0149-7634(94)00021-R 7770196

[26] Zhang M Kelley AE Intake of saccharin, salt, and ethanol solutions is increased by infusion of a mu opioid agonist into the nucleus accumbens. Psychopharmacology (2002) 159:415 10.1007/s00213-001-0932-y 11823894

[27] Mysels DJ Sullivan MA The relationship between opioid and sugar intake: review of evidence and clinical applications. J Opioid Manag (2010) 6:445 10.5055/jom.2010.0043 21269006 PMC3109725

[28] Castro DC Berridge KC Opioid hedonic hotspot in nucleus accumbens shell: mu, delta, and kappa maps for enhancement of sweetness “liking” and “wanting”. J Neurosci (2014) 34:4239–50. 10.1523/JNEUROSCI.4458-13.2014 24647944 PMC3960467

[29] Eikemo M Løseth GE Johnstone T Gjerstad J Willoch F Leknes S. Sweet taste pleasantness is modulated by morphine and naltrexone. Psychopharmacology (2016) 233:3711–23. 10.1007/s00213-016-4403-x 27538675

[30] Martell BA O’Connor PG Kerns RD Becker WC Morales KH Kosten TR Systematic review: opioid treatment for chronic back pain: prevalence, efficacy, and association with addiction. Ann Intern Med (2007) 146:116–27. 10.7326/0003-4819-146-2-200701160-00006 17227935

[31] Sehgal N Manchikanti L Smith HS. Prescription opioid abuse in chronic pain: a review of opioid abuse predictors and strategies to curb opioid abuse. Pain Physician (2012) 15:ES67–92. 22786463

[32] Huang P Kehner GB Cowan A Liu-Chen LY. Comparison of pharmacological activities of buprenorphine and norbuprenorphine: norbuprenorphine is a potent opioid agonist. J Pharmacol Exp Ther (2001) 297:688–95. 11303059

[33] Carroll ME Lac ST. Effects of buprenorphine on self-administration of cocaine and a nondrug reinforcer in rats. Psychopharmacology (1992) 106:439–46. 10.1007/BF02244812 1579619

[34] Sorge RE Stewart J. The effects of chronic buprenorphine on intake of heroin and cocaine in rats and its effects on nucleus accumbens dopamine levels during self-administration. Psychopharmacology (2006) 188:28–41. 10.1007/s00213-006-0485-1 16902770

[35] Mello NK Lukas SE Kamien JB Mendelson JH Drieze J Cone EJ The effects of chronic buprenorphine treatment on cocaine and food self-administration by rhesus-monkeys. J Pharmacol Exp Ther (1992) 260:1185–93. 1545386

[36] Montoya ID Gorelick DA Preston KL Schroeder JR Umbricht A Cheskin LJ Randomized trial of buprenorphine for treatment of concurrent opiate and cocaine dependence. Clin Pharmacol Ther (2004) 75:34–48. 10.1016/j.clpt.2003.09.004 14749690 PMC2633656

[37] Kosten TR Rosen MI Schottenfeld R Ziedonis D. Buprenorphine for cocaine and opiate dependence. Psychopharmacol Bull (1992) 28:15–9. 1609037

[38] Schottenfeld RS Pakes J Ziedonis D Kosten TR. Buprenorphine: dose-related effects on cocaine and opioid use in cocaine-abusing opioid-dependent humans. Biol Psychiatry (1993) 34:66–74. 10.1016/0006-3223(93)90258-F 8373940

[39] Greenwald MK Schuh KJ Stine SM. Transferring methadone-maintained outpatients to the buprenorphine sublingual tablet: a preliminary study. Am J Addict (2003) 12:365–74. 10.1111/j.1521-0391.2003.tb00550.x 14504028

[40] Lutfy K Eitan S Bryant CD Yang YC Saliminejad N Walwyn W Buprenorphine-induced antinociception is mediated by mu-opioid receptors and compromised by concomitant activation of opioid receptor-like receptors. J Neurosci (2003) 23:10331–7. 14614092 10.1523/JNEUROSCI.23-32-10331.2003 PMC6741014

[41] Khroyan TV Polgar WE Orduna J Montenegro J Jiang F Zaveri NT Differential effects of Nociceptin/Orphanin FQ (NOP) receptor agonists in acute versus chronic pain: studies with bifunctional NOP/mu receptor agonists in the sciatic nerve ligation chronic pain model in mice. J Pharmacol Exp Ther (2011) 339:687–93. 10.1124/jpet.111.184663 21859931 PMC3199991

[42] Reid LD Glick SD Menkens KA French ED Bilsky EJ Porreca F Cocaine self-administration and naltrindole, a delta-selective opioid antagonist. Neuroreport (1995) 6:1409–12. 10.1097/00001756-199507100-00012 7488736

[43] Beardsley PM Howard JL Shelton KL Carroll FI. Differential effects of the novel kappa opioid receptor antagonist, JDTic, on reinstatement of cocaine-seeking induced by footshock stressors vs cocaine primes and its antidepressant-like effects in rats. Psychopharmacology (2005) 183:118–26. 10.1007/s00213-005-0167-4 16184376

[44] McLaughlin JP Land BB Li S Pintar JE Chavkin C. Prior activation of kappa opioid receptors by U50, 488 mimics repeated forced swim stress to potentiate cocaine place preference conditioning. Neuropsychopharmacology (2006) 31:787–94. 10.1038/sj.npp.1300860 16123754 PMC2096772

[45] Valdez GR Platt DM Rowlett JK Rüedi-Bettschen D Spealman RD κ Agonist-induced reinstatement of cocaine seeking in squirrel monkeys: a role for opioid and stress-related mechanisms. J Pharmacol Exp Ther (2007) 323:525–33. 10.1124/jpet.107.125484 17702903

[46] Redila VA Chavkin C. Stress-induced reinstatement of cocaine seeking is mediated by the kappa opioid system. Psychopharmacology (2008) 200:59–70. 10.1007/s00213-008-1122-y 18575850 PMC2680147

[47] Bruchas M Land B Chavkin C. The dynorphin/kappa opioid system as a modulator of stress-induced and pro-addictive behaviors. Brain Res (2010) 1314:44–55. 10.1016/j.brainres.2009.08.062 19716811 PMC2819621

[48] Van’t Veer A Carlezon WA. Role of kappa-opioid receptors in stress and anxiety-related behavior. Psychopharmacology (2013) 229:435–52. 10.1007/s00213-013-3195-5 23836029 PMC3770816

[49] Funk D Coen K Lê A The role of kappa opioid receptors in stress-induced reinstatement of alcohol seeking in rats. Brain Behav (2014) 4:356–67. 10.1002/brb3.222 24944865 PMC4055186

[50] Grella SL Funk D Coen K Li Z Lê A. Role of the kappa-opioid receptor system in stress-induced reinstatement of nicotine seeking in rats. Behav Brain Res (2014) 265:188–97. 10.1016/j.bbr.2014.02.029 24583188 PMC4082245

[51] Schindler AG Li S Chavkin C Behavioral stress may increase the rewarding valence of cocaine-associated cues through a dynorphin/κ-opioid receptor-mediated mechanism without affecting associative learning or memory retrieval mechanisms. Neuropsychopharmacology (2010) 35:1932–42. 10.1038/npp.2010.67 20445500 PMC2904851

[52] Al-Hasani R Mccall JG Foshage AM Bruchas MR. Locus coeruleus kappa-opioid receptors modulate reinstatement of cocaine place preference through a noradrenergic mechanism. Neuropsychopharmacology (2013) 38:2484–97. 10.1038/npp.2013.151 23787819 PMC3799068

[53] Negus SS. Effects of the kappa opioid agonist U50, 488 and the kappa opioid antagonist nor-binaltorphimine on choice between cocaine and food in rhesus monkeys. Psychopharmacology (2004) 176:204–13. 10.1007/s00213-004-1878-7 15112031

[54] Pfeiffer A Brantl V Herz A Emrich HM Psychotomimesis mediated by k opiate receptors. Science (1986) 233:774–7. 10.1126/science.3016896 3016896

[55] Millan MJ. Kappa-opioid receptors and analgesia. Trends Pharmacol Sci (1990) 11:70–6. 10.1016/0165-6147(90)90321-X 2156363

[56] Sante A Nobre M Brandao M Place aversion induced by blockade of µ or activation of κ opioid receptors in the dorsal periaqueductal gray matter. Behav Pharmacol (2000) 11:583–9. 10.1097/00008877-200011000-00005 11198129

[57] Anderson RI Morales M Spear LP Varlinskaya EI. Pharmacological activation of kappa opioid receptors: aversive effects in adolescent and adult male rats. Psychopharmacology (2014) 231:1687–93. 10.1007/s00213-013-3095-8 23604334 PMC3760984

[58] Ehrich JM Messinger DI Knakal CR Kuhar JR Schattauer SS Bruchas MR Kappa opioid receptor-induced aversion requires p38 MAPK activation in VTA dopamine neurons. J Neurosci (2015) 35:12917–31. 10.1523/JNEUROSCI.2444-15.2015 26377476 PMC4571610

[59] Freye E Latasch L Development of opioid tolerance—molecular mechanisms and clinical consequences. Anasthesiol Intensivmed Notfallmed Schmerzther (2003) 38:14–26. 10.1055/s-2003-36558 12522725

[60] Jamison RN Mao JR. Opioid analgesics. Mayo Clin Proc (2015) 90:957–68. 10.1016/j.mayocp.2015.04.010 26141334

[61] Tian JH Zhang W Fang Y Xu W Grandy DK Han JS. Endogenous orphanin FQ: evidence for a role in the modulation of electroacupuncture analgesia and the development of tolerance to analgesia produced by morphine and electroacupuncture. Br J Pharmacol (1998) 124:21–6. 10.1038/sj.bjp.0701788 9630338 PMC1565350

[62] Ozawa A Brunori G Mercatelli D Wu J Cippitelli A Zou B Knock-in mice with NOP-eGFP receptors identify receptor cellular and regional localization. J Neurosci (2015) 35:11682–93. 10.1523/JNEUROSCI.5122-14.2015 26290245 PMC4540802

[63] Rizzi A Cerlesi MC Ruzza C Malfacini D Ferrari F Bianco S Pharmacological characterization of cebranopadol a novel analgesic acting as mixed Nociceptin/Orphanin FQ and opioid receptor agonist. Pharmacol Res Perspect (2016) 4:e00247. 10.1002/prp2.247 28116100 PMC5242173

[64] Rajagopal S Shenoy SK. GPCR desensitization: acute and prolonged phases. Cell Signal (2017). 10.1016/j.cellsig.2017.01.024 28137506 PMC5533627

